# Factors Influencing Primary Care Access for Common Mental Health Conditions Among Adults in West Africa: Protocol for a Scoping Review

**DOI:** 10.2196/58890

**Published:** 2024-10-02

**Authors:** Nhyira Yaw Adjei-Banuah, Roberta Naa Barkey Ayiku, Veronika Reichenberger, David Sasu, Tolib Mirzoev, Adrianna Murphy, Sammy Ohene, Edward Antwi, Irene Akua Agyepong

**Affiliations:** 1 Ghana College of Physicians and Surgeons Accra Ghana; 2 Department of Global Health and Development London School of Hygiene and Tropical Medicine London United Kingdom; 3 Department of Computer Science and Information Systems Ashesi University Berekuso Ghana; 4 Department of Health Services Research and Policy London School of Hygiene and Tropical Medicine London United Kingdom; 5 School of Medicine University of Ghana Accra Ghana

**Keywords:** scoping review, mental health, noncommunicable disease, primary care, access, barriers, enablers

## Abstract

**Background:**

Mental health conditions are expressed in various ways in different people, and access to health care for these conditions is affected by individual factors, health care provider factors, and contextual factors. These factors may be enablers or barriers to accessing primary care for mental health conditions. Studies have reported a gap in treatment for mental health conditions in many countries in West Africa due to barriers along the access pathway. However, to the best of our knowledge, there is yet to be a review of the factors influencing access to primary care for common mental health conditions among adults in West Africa.

**Objective:**

Our scoping review will explore the factors influencing access to primary care for common mental health conditions among adults aged 18 years and older in West Africa from 2002 to 2024.

**Methods:**

Our review will follow the approach to scoping reviews developed by Arksey and O’Malley in 2005. This approach has five stages: (1) identifying the research question; (2) identifying relevant studies; (3) selecting studies; (4) charting the data; and (5) collating, summarizing, and reporting the results. We will search electronic databases (PubMed, Embase, PsycINFO, Cairn.info, and Google Scholar), source gray literature from relevant websites (the World Health Organization and country-specific websites), and manually explore reference lists of relevant studies to identify eligible records. Pairs of independent authors (NYA-B, RNBA, VR, or DS) will screen the titles, abstracts, and full texts of studies based on predefined eligibility criteria. We will use a data extraction tool adopted from the *JBI Manual for Evidence Synthesis* to chart the data. Deductive, thematic analysis will be used to categorize factors influencing access to mental health care under predetermined themes. New themes derived from the literature will also be charted.

**Results:**

Database searches were conducted between February 1, 2024, and February 12, 2024. As of July 2024, the review report is being drafted, and it will be disseminated through publication in a peer-reviewed journal.

**Conclusions:**

The results of the review will inform decision-making on policies, programs, and their implementation in West Africa to improve primary care access for mental health care.

**International Registered Report Identifier (IRRID):**

DERR1-10.2196/58890

## Introduction

### Background

Mental health is defined as “a state of mental well-being that enables people to cope with the stresses of life, realize their abilities, learn well and work well, and contribute to their community. It is an integral component of health and well-being, and is more than the absence of a mental disorder” [1]. It comprises an intricate range of mental health states, and not simply the absence of a defined mental disorder. Mental health conditions are expressed in various ways in different people and are affected by numerous individual and environmental factors [1]. Some common mental health conditions include depression, anxiety disorders, bipolar disorder, and schizophrenia [1].

The global figures on mental health conditions are staggering. In 2019, it was estimated that 970 million people were living with a mental condition, 82% of whom were living in a low- or middle-income country (LMIC) [2]. In that same year, 301 million people were said to be living with anxiety disorders, while 280 million people had depressive disorders globally [2]. Furthermore, mental disorders are the foremost cause of years lived with disability (YLDs), contributing to 1 in 6 YLDs worldwide [2]. In 2019, 5.6% of YLDs were attributable to depression alone [2]. A scoping review of population-based epidemiological studies in Africa conducted in 2020 revealed that the prevalence of mental disorders ranged from 3.3% to 9.8% for mood disorders, 5.7% to 15.8% for anxiety disorders, and 3.7% to 13.3% for substance use disorders [3].

Apart from the alarmingly high prevalence of various mental disorders, an emerging area of concern is the link between these disorders and noncommunicable diseases (NCDs) such as hypertension and diabetes. Reviews and meta-analyses have established a connection between mental disorders and cardiovascular diseases [4,5]. Primary studies conducted in LMICs have confirmed a similar association between mental disorders and NCDs [6-8]. A study conducted by Nkporbu et al [9] in Nigeria to determine the pattern and prevalence of psychiatric comorbidity among patients with hypertension revealed that almost two-thirds of the 232 participants had coexisting mental disease, most commonly depression, followed by anxiety disorders. These findings are consistent with that of a similar study by Keskin and Bilge [8] in a primary care setting in Ankara, Turkey, which found that about half of the patients with hypertension and diabetes in their study had mental health disorders, the most frequent of which were mood disorders.

Common mental disorders are a collection of illnesses characterized by depression, anxiety, and the experience of unexplained bodily symptoms [10]. On the other hand, mood disorders are psychiatric conditions characterized by prolonged and marked emotional disturbance [11]. While persons living with NCDs are at risk of some mental disorders, mood disorders are their most commonly encountered mental disorders [8,9]. For instance, depressive illnesses are frequent among patients with diabetes [6]. People with chronic NCDs may experience depression due to challenges with managing their condition [12]. The World Health Organization (WHO) recommends a protocol for managing mental conditions in primary care settings. This means that people with physical conditions (including NCDs) need to be screened for mental disorders (by appropriate history taking and mental state examination where needed) and offered the required psychosocial (psychoeducation, counseling, promotion of function, and psychological treatment) or pharmacological intervention as indicated [13].

The WHO defines primary care as “a model of care that supports first-contact, accessible, continuous, comprehensive and coordinated person-focused care” [14]. In health care, access can be defined as “the opportunity to have healthcare needs fulfilled” [15]. Despite the prevalence of mental health conditions and the emphasis on access to primary care, the West African subregion has responded slowly to the burden of these disorders. Mental health policies and legislation in most West African countries are either obsolete or have major pitfalls, budgetary allocation for mental health is insufficient, and mental health services are mostly limited to large facilities that are poorly distributed geographically, thereby impeding access [16]. In addition, some people believe mental illnesses are caused by evil spirits [17]. Consequently, in West Africa, patients seek primary care at allopathic facilities as well as from alternative health providers such as herbal medicine practitioners [16]. All these avenues of first-contact care provide primary care for mental health conditions.

Providing timely and accessible care for mental health conditions requires the efficient integration of mental health care into existing primary care systems [18]. This ensures that people with mental illnesses are provided with care within their community that is adapted to fit their context [19]. However, in many West African countries, this is not the case. For instance, in Ghana, although there have been efforts to provide adequate mental health care at the primary care level, it has been fraught with challenges with personnel, policy implementation, and funding; as such, persons with mental illnesses have to travel long distances to specialized hospitals to receive care [20]. The situation is similar in countries such as Liberia, Sierra Leone, and the Gambia, where mental health care provision at the primary care level is almost nonexistent [16]. Also, in Nigeria, most people with mental health challenges do not have access to care in their communities. Instead, they have to resort to the few overburdened psychiatric facilities for care, regardless of illness severity [21].

As a result, mental health care provision in West Africa is largely pluralistic, characterized by a complex, uncoordinated system of allopathic, traditional, complementary, and alternative medicine providers [22-26]. In Burkina Faso, most patients seen at formal mental health care facilities will have already visited a traditional medicine practitioner for some form of treatment [22]. Similarly, in Ghana, Nigeria, and Liberia, it is common for service users to seek mental health care from traditional healers, faith healers, and other alternative medicine providers before eventually resorting to formal mental health care [24-26]. Rather than benefiting from the collaborative effort of different types of mental health service providers, people with mental health challenges in West Africa experience delays in receiving adequate primary health care due to a lack of an efficient coordination system [26].

Factors influencing primary health care access for common mental health conditions can be broadly categorized as factors that act as barriers to access and factors that act as enablers to access. Factors we identified in the literature that can act as barriers to primary care access for common mental health conditions include the stigmatization of mental health service users [20,27-36]. Also, a lack of knowledge of mental health on the part of patients and the absence of capacity-strengthening programs for service providers hinder effective primary care for mental disorders [20,27,32,33,36-38]. Furthermore, individual experiences of mental health services such as the perceived ineffectiveness of some medications and unpleasant side effects could serve as barriers [39]. As in other LMICs, costs of care, including medication and transportation, also hinder people from seeking mental health care in West Africa [17,20,30-32,34,40,41]. This represents a significant burden to the families of service users [20].

A study in Liberia by Herman et al [41] revealed that some service users and their families do not seek help because they live too far from health facilities. Some persons in Nigeria also report prolonged waiting times at mental health facilities [32]. In many West African countries, mental health services are centralized despite the existence of decentralization and integration policies [28,42]. Moreover, there are many bottlenecks with the referral systems, aggravated by the lack of application of policies on task-shifting —the process of delegating tasks from specialized health workers to other health workers—in mental health care delivery [40,43,44]. Also, primary health care facilities frequently run out of essential medications for treating mental health disorders [34,40,45]. This is usually reported simultaneously with the poor state of psychiatric facilities, hindering quality mental health care provision [40,45]. Different studies also identify poor human resource management as a major hindering factor. There are not enough mental health workers [34,40,45]. The few available also need further training to improve their service delivery.

Factors that act as enablers of access to primary mental health care identified in the literature are, first, support services for persons with mental health conditions [20]. Such services are predominantly provided by the families of those affected [20]. Religious organizations, neighbors, and friends also provide support in certain areas [20]. For example, in the 5 northernmost administrative regions of Ghana, there are self-help groups that provide social and financial support to service users and caregivers [46]. Second, the existence of mental health laws is an enabling factor [45]. In some instances, such as in the Gambia, there is mental health legislation in place, although it is not backed by law [36]. Finally, although its implementation has been met with some challenges, studies have shown that task-shifting enhances the provision of quality mental health care delivery [28,43,47].

### Rationale

Despite the need and importance to inform policy and program decision-making for the selection, design, and implementation of interventions to address and improve mental health in West Africa, we did not find a systematic review of these enablers and barriers or factors that influence primary health care access for mental health in West Africa. A scoping review of population-based epidemiological studies of mental disorders in Africa was conducted [3], but it was a prevalence study that did not address the factors that influence access to primary care for these conditions. An integrative review of enablers and barriers to accessing mental health services was limited to Ghana [20], 1 of the 15 countries of the Economic Community of West African States (ECOWAS). Similar reviews were not available for the remaining countries of ECOWAS. A systematic review of the literature on factors influencing primary mental health care access will also help to identify gaps in the literature, which will necessitate further research to inform policy and program decision-making. However, it was uncertain from our initial literature review whether the breadth and depth of the literature available across the 15 West African countries that made up ECOWAS at the time of the review was adequate for a classical systematic review. We therefore chose to start with a scoping review. Scoping reviews are a form of systematic review that is particularly useful in situations of uncertainties about the breadth and depth of the literature. They enable systematic mapping of the available evidence and are particularly suitable for identifying knowledge gaps and the main sources of evidence on a concept in these situations of uncertainty about the evidence base [48].

### Objectives

The main objective of this review is to identify factors influencing access to primary care for common mental health conditions (depression, bipolar disorders, schizophrenia, anxiety disorders, stress disorders, and substance use disorders) in adults aged 18 years and older in West Africa from 2002 to 2024.

The specific objectives, all related to access to primary care for common mental health conditions among adults aged 18 years and older in West Africa from 2002 to 2024, are as follows: (1) to identify patient-level factors that affect and how they affect access to care; (2) to identify primary health care facility–level factors that affect and how they affect access to care; (3) to identify specific contextual factors (societal beliefs, norms, and practices) that affect and how they affect access to care; and (4) to synthesize implications for co-design with stakeholders in interventions for primary mental health care, identify gaps in the literature, and suggest areas for further research into primary care for common mental health conditions among adults aged 18 years and older in West Africa from 2002 to 2024.

### Conceptual Framework for Enablers and Barriers to Primary Care Access for Mental Health

We adapted a conceptual framework to guide our exploration and analysis of factors influencing access to primary care for mental health, drawing on the work of Levesque et al [15] and the concept of context from the framework of context described by Leichter [49].

**Figure 1:**
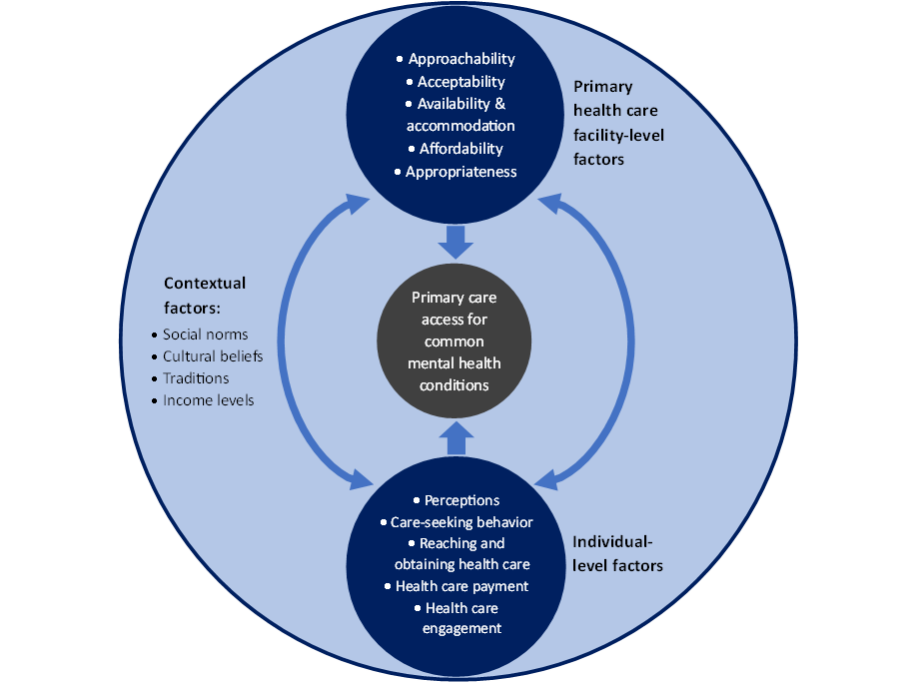
Conceptual framework for access to primary care.

The framework (Figure 1) categorizes and explores factors (or enablers and barriers) that influence access to primary care for mental health, further categorized into factors that operate at the level of individual perspectives and the level of the primary health care facility. Enablers and barriers influencing access to care related to the primary health care facility include (1) approachability, (2) acceptability, (3) availability and accommodation, (4) affordability, and (5) appropriateness of services. Enablers and barriers influencing access to care related to the individual include (1) perceptions of care, (2) care-seeking behavior, (3) reaching and obtaining health care, (4) health care payment, and (5) health care engagement.

The framework also includes specific contextual factors that influence access to primary care for mental health conditions. We defined contextual factors as those elements outside individuals but within their environment that interact with the individual and influence their approach to seeking and using health care services [50]. Thus, although Leichter [49] subdivided contextual factors into situational, structural, cultural, and environmental factors, we only focused on the cultural factors to meet our third specific objective. These contextual factors include sociocultural and socioeconomic factors such as social norms, cultural beliefs, traditions, and income levels [50]. These sociocultural and socioeconomic enablers and barriers act as background factors in the society of the individual and primary health care facility that shape other enablers and barriers to accessing primary care for mental health.

Individuals’ access to primary care for mental health will depend on their perceptions of care, their care-seeking behavior, their ability to reach a primary care facility, and their capacity to pay for and engage with the available services. Access will also depend on how approachable the primary care facility is, how well the individual accepts the mode of service delivery, the services available, how affordable they are, and how appropriate they are for attending to mental health. These factors are all affected by social constructs of mental health such as the beliefs, norms, traditions, and income levels of that specific society. Therefore, primary care access for common mental health conditions among adults in West Africa is affected by interactions between enablers and barriers to access at the individual and primary health care facility level, and these interactions take place within a background of, and are shaped by, specific contextual factors.

## Methods

### Protocol Design

Our review will follow the approach developed by Arksey and O’Malley [51] in 2005. Their seminal work in scoping reviews was, in many respects, a foundation for further initiatives to bring some standardization to the structure of scoping reviews. As they suggested, we will follow these five stages: (1) identifying the research question; (2) identifying relevant studies; (3) selecting studies; (4) charting the data; and (5) collating, summarizing, and reporting the results. We will not assess the risk of bias or quality of the studies included, as this scoping review seeks to present an overview of the information available on the research topic.

### Identifying the Research Question

The research question was developed based on the gaps identified from the initial literature review and in consultation with a team of researchers with expertise in the field. Following an iterative process, the main research question we seek to answer is as follows: “What factors influence access to primary care for common mental health conditions in adults aged 18 years and older in West Africa?”

As defined earlier, common mental conditions are a collection of disorders characterized by depression, anxiety, and the experience of unexplained bodily symptoms [10]. The mental conditions of interest to our review are depression, bipolar disorders, schizophrenia, anxiety disorders, stress disorders, and substance use disorders.

### Identifying Relevant Studies

#### Eligibility Criteria

We will focus on studies published between January 1, 2002, and January 31, 2024, and will use the participants, concept, context, and type of study (PCCS) framework to identify studies that are eligible for our review.

#### Participants

Studies in which participants are adults aged 18 years or older who have a mental disorder will be considered. These patients should be those who seek primary care at various facilities. Studies that report the perspective of other stakeholders, such as health care workers (including allopathic and alternative medicine providers), policy makers, and caregivers (relatives and support workers) of these patients, will be included. We will use the definition of primary care stated in the Background section of this paper. Studies conducted in adults and children in which the data for participants aged 18 years or older cannot be extracted separately will be excluded. Studies conducted on pregnant women will also be excluded.

#### Concept

We are interested in studies reporting factors influencing primary care of common mental health conditions in adults. These could either be individual characteristics or behaviors such as income, livelihood, or gender; they could also emanate from environmental and contextual influences such as societal beliefs, norms, and practices. The mental health conditions we will consider include depression, bipolar disorders, schizophrenia, anxiety disorders, stress disorders, and substance use disorders in the study participants.

#### Context

The included studies had to be conducted in 1 or more countries in West Africa. The 15 countries that belonged to ECOWAS over the period covered by this study were Benin, Burkina Faso, Cabo Verde, Cote d’Ivoire, the Gambia, Ghana, Guinea, Guinea-Bissau, Liberia, Mali, Niger, Nigeria, Senegal, Sierra Leone, and Togo. Studies conducted in settings that include West African countries and other countries in which the data for the West African countries cannot be extracted separately will be excluded.

#### Type of Study

Primary studies, whether experimental or nonexperimental and descriptive or analytical, will be considered for our scoping review. Systematic and scoping reviews that meet other inclusion criteria will also be considered. Unpublished studies and gray literature reporting data that answer our research question will also be included. On the other hand, case studies, case reports, and anecdotal evidence from gray literature will be excluded.

### Conducting the Search

The search will include studies conducted from January 1, 2002, to January 31, 2024. The start date was chosen because that was when the WHO, through the Mental Health Gap Action Programme (mhGAP) [52], spearheaded a concerted effort to address the global burden of mental health conditions, with particular attention paid to LMICs. Under this initiative, the WHO, through partnerships with member states, outlined a comprehensive strategy to strengthen the capacity of governments to reduce the risk and burden of mental health conditions [52]. Our search will be restricted to English and French, the official languages of most countries in West Africa. The databases we will search will include PubMed, Embase, PsycINFO, Cairn.info, and Google Scholar. Key concepts from our research question and alternative terms (Table 1) will be used to develop a full search strategy. The search strategy for PubMed is provided in Multimedia Appendix 1. This search strategy will then be adapted for each database. The coauthors will search the databases with the help of an information scientist. The bibliographies of studies included in the initial search will be manually explored for additional papers. The search results will then be exported to Rayyan, a web-based screening tool, which will be used to screen the titles and abstracts. Records that are in French will be translated with the help of a bilingual secretary and translation software (DeepL Translate; DeepL SE).

**Table 1:** Key concepts and alternative terms.

Concepts	Terms
Mental health condition	common mental disorder; mental disorder; mental illness; stress; stress disorder; anxiety; anxiety disorder; generalized anxiety disorder; depression; recurrent depression; major depression; major depressive illness; depressive illness; depressive disorder; mood disorder; substance abuse; substance use disorder; bipolar disorder; schizophrenia
Hypertension	high blood pressure; raised blood pressure; increased blood pressure; hypertensive disease; hypertensives; hypertensive patients
Diabetes	diabetes mellitus; high blood glucose; high plasma glucose; raised blood glucose; increased blood glucose; diabetics; diabetic patients
Influence	barrier; hindrance; hinder; obstacle; difficult*; obstruct; prevent; limit*; restrain*; inhibit; enable*; facilitat*; support; opportunit*; aid; ease; promot*; help
West Africa	Benin; Burkina Faso; Cape Verde; Cabo Verde; Cote d’Ivoire; Gambia; Ghana; Guinea; Guinea-Bissau; Ivory Coast; Liberia; Mali; Niger; Nigeria; Sierra Leone; Senegal; Togo
Primary care	health education; patient counseling; psychotherapy; exercise; cognitive behavioural therapy; behavioural therapy; stress therapy; selective serotonin reuptake inhibitors; SSRIs; tricyclic antidepressants; TCAs; antidepressants; anxiolytics; rehabilitation; primary care; primary healthcare; health services; healthcare

### Study Selection

**Figure 2:**
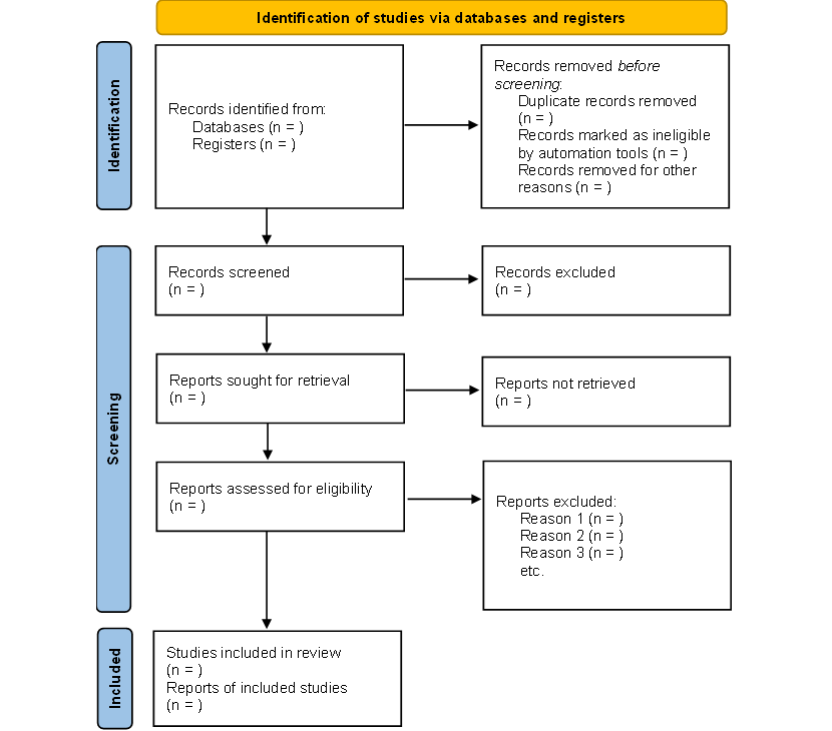
PRISMA flow diagram.

Rayyan will be used to handle the results, remove duplicates, and screen the records. Pairs of reviewers (NYA-B, RNBA, VR, or DS) will independently screen the titles and abstracts of the studies. We will then select the studies that are relevant to our topic. After this, we will retrieve and read the full text of the selected studies. Studies that meet the inclusion criteria described above will be selected for final analysis. In our final report, we will document the reasons for excluding any study. The operationalization of our eligibility criteria will be tested beforehand, using a random sample of 5 of the retrieved studies. This will be done by the pairs of independent reviewers to ensure reliability. A third reviewer will settle any disagreements following this process. The results of the entire search strategy and study selection process will be illustrated in the final report using the Preferred Reporting Items for Systematic Reviews and Meta-Analyses (PRISMA) [53] flow diagram, shown in Figure 2. Zotero (Corporation for Digital Scholarship), a reference management tool, will be used to add citations and a list of references in the final report.

### Charting the Data

**Figure 3:**
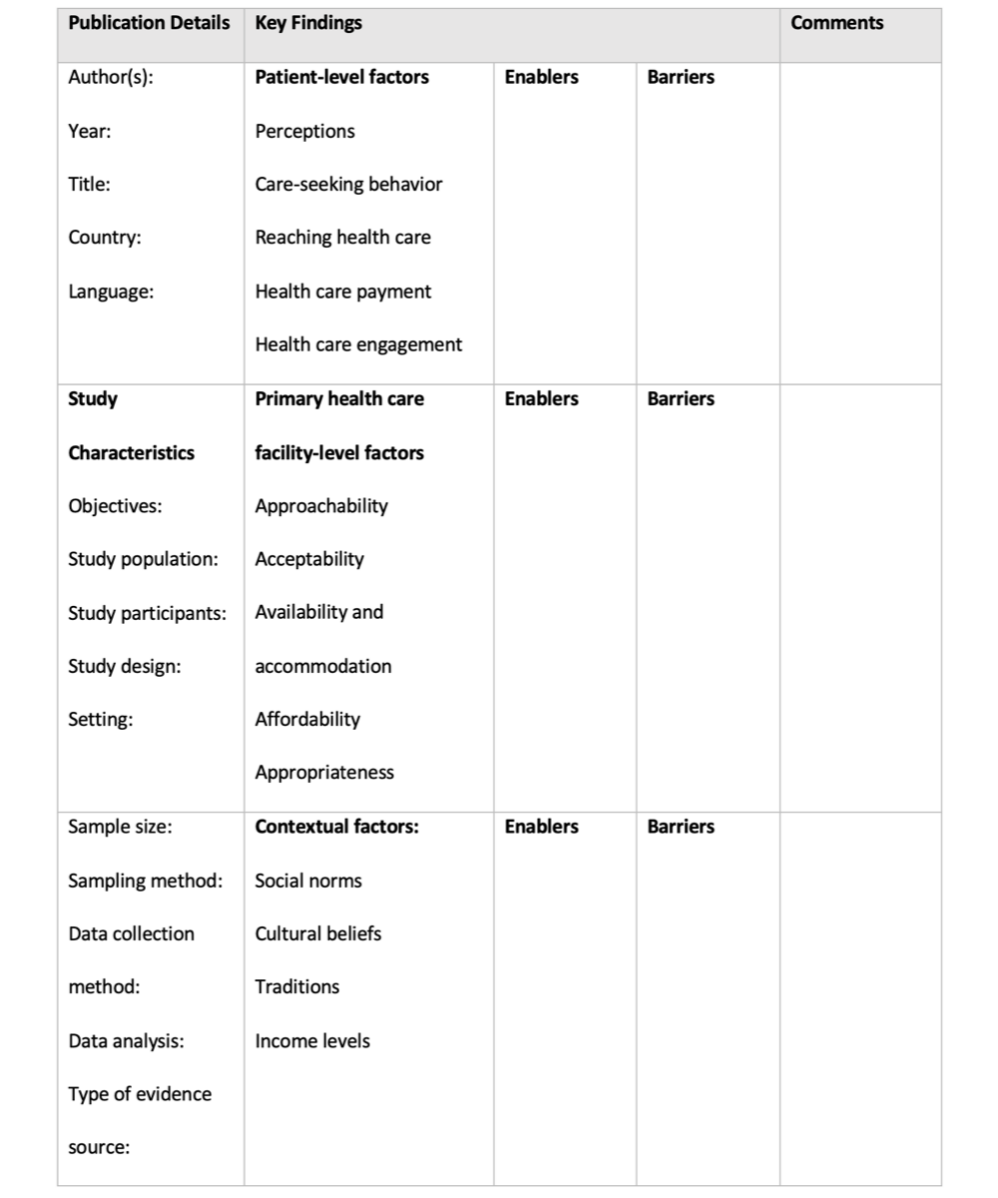
Draft data extraction tool.

The data will be charted using Microsoft Excel spreadsheets. A draft data extraction tool adopted from the *JBI Manual for Evidence Synthesis* [54] will be used (Figure 3). The data to be extracted will include publication details (author, year of publication, title, country, and language), study characteristics (objectives; characteristics of the study population and participants; sample characteristics, including sample size and sampling method; study design; and type of evidence source‚ whether it is a review, primary study, or website), the context, the key findings, and comments such as strengths or weaknesses of the study. The key findings will include the results of the study as well as information related to the objectives of our review. Pairs of independent reviewers will pilot the tool on a sample of 5 studies to assess validity. Where necessary, modifications will be made to the tool and documented in the final review. A third independent reviewer will settle any disagreements. We will contact the authors for more information and clarification concerning missing data.

### Collating, Summarizing, and Reporting the Results

The extracted data will be summarized and presented narratively according to the framework for access to primary care described earlier. Based on this, the following themes determined a priori will be used to categorize the influencing factors: perceptions; care-seeking behavior; reaching and obtaining health care; health care payment; health care engagement; approachability, acceptability, availability and accommodation, affordability, and appropriateness; and social norms, cultural beliefs, traditions, and income levels. Under each theme, the factors will be identified as enablers or barriers to primary care access. The level at which these factors act, be it patient level, primary health care facility level, or contextual (beliefs, norms, and practices), will also be presented. Additionally, new themes derived from the literature will be organized as subthemes under the predefined domains in the framework. Themes that do not fit under any of the predefined domains will be added as new main themes. The various themes and subthemes will be illustrated using appropriate diagrams and tables. Subgroup comparisons will be done where relevant. Subgroups may include gender, age groups, social status, and other relevant subgroups identified in the studies. Inconsistencies and gaps in the literature will also be identified, with recommendations made for further research to fill those gaps.

## Results

Database searches were conducted between February 1, 2024, and February 12, 2024. After screening, 28 papers were included in the final analysis. As of July 2024, the review report was being drafted and will be disseminated through publication in a peer-reviewed journal.

## Discussion

### Principal Findings

This study will gather available evidence on the enablers and barriers to accessing primary care for common mental health conditions in West Africa. Findings will be related to perceptions, care-seeking behavior, reaching and obtaining health care, health care payment, and health care engagement of the individual. Findings will also include approachability, accessibility, availability and accommodation, affordability, and appropriateness of primary health care services for mental health. Our review is likely to discover that these enablers and barriers to access are influenced by the sociocultural and economic contexts of West African countries within which people with mental illnesses seek care.

The proposed review is a part of the first phase of the Stop-NCD project, which aims to improve the health and well-being of populations in West Africa by developing the capacity for high-quality research to inform improved prevention, diagnosis, and treatment of interconnected NCDs (hypertension, diabetes, and coexisting mental disorders). The project also seeks to understand mental health disorders in relation to people with hypertension and diabetes. This is the reason for our decision to include the interconnection among mental health conditions, hypertension, and diabetes in our search strategy, though it is not part of the main objectives for our review.

### Comparison to Prior Work

As mentioned earlier, to the best of our knowledge this is the first review of the factors influencing primary care access for mental health conditions among adults in West Africa. However, similar studies have been conducted in Southeast Asia, as well as in Australia and other high-income countries [55-57]. In these reviews, self-awareness, resources and information, affordability, accessibility, patient-centered care, use of technology, mental health literacy, culturally sensitive practice, and gender were reported as factors that influence access to mental health care among adults in these regions [55-57]. In our review report, our study findings will be compared to these findings and the results of other identified studies.

### Strengths

To the best of our knowledge, this will be the first scoping review to map the factors that influence primary care for mental conditions in adults in West Africa. Furthermore, our review protocol follows the PRISMA Extension for Scoping Reviews (PRISMA-ScR) guidelines [58]. Also, the review will use a comprehensive search strategy for eligible studies that will include 5 electronic databases, key websites for gray literature, and reference lists of pertinent studies.

### Limitation

We perceive a potential challenge in accessing eligible papers reported in French. However, we believe that using the services of a bilingual secretary and using DeepL to assist us with translation will help us overcome this challenge.

### Future Directions

Considering the similarities in the socioeconomic and political climate among most countries in sub-Saharan Africa, the findings from our review, though focused on West Africa, will be useful to various actors in other countries in sub-Saharan Africa who seek to answer a similar question. The findings from our review will also highlight specific areas where further research is needed.

### Conclusions

Exploring the barriers and enablers to accessing primary care for common mental health conditions is essential to shape policies to address the pressing need for quality and accessible mental health care in this subregion.

## Appendix

Multimedia Appendix 1 Search strategy for PubMed.

## Abbreviations

ECOWAS: Economic Community of West African States
LMIC: low- or middle-income country
NCD: noncommunicable disease
PCCS: participants, concept, context, and type of study
PRISMA-ScR: Preferred Reporting Items for Systematic Reviews And Meta-Analyses Extension for Scoping Reviews
PRISMA: Preferred Reporting Items for Systematic Reviews and Meta-Analyses
WHO: World Health Organization
YLD: year lived with disability

## Data Availability

Data sharing is not applicable to this article as no data sets were generated or analyzed during this study.
