# Supplementary material for: Factors Influencing Primary Care Access for Common Mental Health Conditions Among Adults in West Africa: Protocol for a Scoping Review
Source: JMIR Res Protoc. 2024 Oct 2;13:e58890. doi: 10.2196/58890 (PMC11483259; doi:10.2196/58890)
Supplement: Multimedia Appendix 1 [file resprot_v13i1e58890_app1.docx]

# Multimedia Appendix 1: Search strategy for PubMed

| **Search No.** | **Search terms** |
| --- | --- |
| #1 | (((((((((((((((((((("Mental health"[MeSH]) OR ("Mental health condition")) OR ("Common mental disorder")) OR ("mental disorders"[MeSH])) OR ("mental illness")) OR (stress)) OR ("stress disorder")) OR (anxiety[MeSH])) OR ("anxiety disorders"[MeSH])) OR (depression[MeSH])) OR ("recurrent depression")) OR ("major depression")) OR ("major depressive illness")) OR ("depressive illness")) OR ("depressive disorder"[MeSH])) OR ("mood disorders"[MeSH])) OR ("Substance abuse")) OR ("Substance use disorder")) OR ("bipolar disorder"[MeSH])) OR (schizophrenia[MeSH])) OR ("generalized anxiety disorder") |
| #2 | ((((((Hypertension[MeSH]) OR ("High blood pressure")) OR ("Raised blood pressure")) OR ("Increased blood pressure")) OR ("Hypertensive disease")) OR (Hypertensive*)) OR ("Hypertensive patient*") |
| #3 | (((((((Diabetes) OR ("Diabetes Mellitus"[MeSH])) OR ("High blood glucose")) OR ("High plasma glucose")) OR ("Raised blood glucose")) OR ("Increased blood glucose")) OR (Diabetic*)) OR ("Diabetic patient*") |
| #4 | ((((((((((Influence) OR (Barrier)) OR (hindrance)) OR (hinder)) OR (obstacle)) OR (difficult*)) OR (obstruct)) OR (prevent)) OR (limit*)) OR (restrain*)) OR (inhibit) |
| #5 | (((((((enable*) OR (facilitat*)) OR (support)) OR (opportunit*)) OR (aid)) OR (ease)) OR (promot*)) OR (help) |
| #6 | ((((((((((((((((((("Health Education") OR ("Patient counseling")) OR (Psychotherapy[MeSH])) OR (Exercise)) OR ("Cognitive behavioral therapy"[MeSH])) OR ("Behavioral therapy")) OR ("Stress therapy")) OR ("Antidepressive agents"[MeSH])) OR ("Selective serotonin reuptake inhibitors")) OR (SSRIs)) OR ("Tricyclic antidepressants")) OR (TCAs)) OR (Antidepressants)) OR ("anti-anxiety agents"[MeSH])) OR (Anxiolytics)) OR (Rehabilitation)) OR ("Primary care")) OR ("Primary healthcare")) OR ("Health services")) OR (Healthcare) |
| #7 | ((((((((((((((((((("West Africa") OR ("Africa, Western"[MeSH])) OR (Benin[MeSH])) OR ("Burkina Faso"[MeSH])) OR ("Cape Verde")) OR ("Cabo Verde"[MeSH])) OR ("Cote d’Ivoire"[MesH])) OR (Gambia[MeSH])) OR (Ghana[MeSH])) OR (Guinea[MeSH])) OR (Guinea-Bissau[MeSH])) OR ("Ivory Coast")) OR (Liberia[MeSH])) OR (Mali[MeSH])) OR (Niger[MeSH])) OR (Nigeria[MeSH])) OR ("Sierra Leone"[MeSH])) OR (Senegal[MeSH])) OR (Togo[MeSH]) |
| #8 | ((((#1) AND (#2)) AND (#3)) AND (#4)) AND (#6) |
| #9 | ((((#1) AND (#2)) AND (#3)) AND (#5)) AND (#6) |
| #10 | (((#1) AND (#2)) AND (#4)) AND (#6) |
| #11 | (((#1) AND (#2)) AND (#5)) AND (#6) |
| #12 | (((#1) AND (#3)) AND (#4)) AND (#6) |
| #13 | (((#1) AND (#3)) AND (#5)) AND (#6) |
| #14 | ((#1) AND (#4)) AND (#6) |
| #15 | ((#1) AND (#5)) AND (#6) |
| #16 | (#8) AND (#7); Filter- Publication Year: 2002-2024 |
| #17 | (#9) AND (#7); Filter- Publication Year: 2002-2024 |
| #18 | (#10) AND (#7); Filter- Publication Year: 2002-2024 |
| #19 | (#11) AND (#7); Filter- Publication Year: 2002-2024 |
| #18 | (#12) AND (#7); Filter- Publication Year: 2002-2024 |
| #19 | (#13) AND (#7); Filter- Publication Year: 2002-2024 |
| #20 | (#14) AND (#7); Filter- Publication Year: 2002-2024 |
| #21 | (#15) AND (#7); Filter- Publication Year: 2002-2024 |
| Final output: #16, #17, #18, #19, #20, #21, #22, #23 | |
